# Comparison of dietary micronutrient intake in PCOS patients with and without metabolic syndrome

**DOI:** 10.1186/s13048-020-00746-0

**Published:** 2021-01-09

**Authors:** Narges Zaeemzadeh, Shahideh Jahanian Sadatmahalleh, Saeideh Ziaei, Anoshirvan Kazemnejad, Maryam Movahedinejad, Azadeh Mottaghi, Neda Mohamadzadeh

**Affiliations:** 1 Department of Reproductive Health and Midwifery, Faculty of Medical Sciences, Tarbiat Modares University, Tehran, Iran; 2 Department of Biostatistics, Faculty of Medical Sciences, Tarbiat Modares University, Tehran, Iran; 3 Research Center for Prevention of Cardiovascular Diseases, Institute of Endocrinology & Metabolism, Iran University of Medical Sciences, Tehran, Iran

**Keywords:** Polycystic ovarian syndrome (PCOS), Metabolic syndrome (MetS), Antioxidants, Dietary intake, Micronutrients

## Abstract

**Background:**

Polycystic ovarian syndrome (PCOS) is the most common endocrine disorder in reproductive-age women. It is one of the risk factors of metabolic syndrome (MetS). These two syndromes have an inflammatory etiologic foundation along with oxidative stress. The present study aimed to compare the dietary intake of antioxidant micronutrients in PCOS women with and without MetS.

**Materials and methods:**

Overall, 42 participants eligible for this nested case control study were selected by the convenience sampling method. The case group included 14 PCOS patients with MetS and the control group included 28 PCOS patients without MetS. The dietary intake assessment of selenium, chromium, zinc, carotenoids, vitamin D and vitamin E was carried out by a 147-item Food Frequency Questionnaire (FFQ). PCOS and MetS were diagnosed using the Rotterdam criteria and NCEP ATP III, respectively. Statistical analysis was performed using SPSS16 software, T-test and Mann Whitney. Significant *P*-value was considered 0.05.

**Results:**

Dietary intake of antioxidant micronutrients (selenium, zinc, chromium, carotenoids and vitamin E) was significantly lower in the PCOS women with MetS than in the control group (*P* < 0.05).

**Conclusion:**

Since the PCOS patients without MetS had more intake of the aforementioned micronutrients than those with MetS, it is assumed that the dietary intake of these nutrients could probably have a protective effect on MetS.

## Introduction

Polycystic ovarian syndrome (PCOS), as the most common endocrine disorder in women of reproductive age, has engaged about 5–10% of women throughout the world [1]. In addition to irregular menstrual cycles, chronic anovulation and hyperandrogenism, numerous metabolic complications are also seen in PCOS-afflicted women such as obesity, hyperlipidemia, hyperinsulinemia, insulin resistance (IR), dysglycemia, and increased risk of cardiac diseases and endometrial cancer [1]. If PCOS is left untreated in the long term, the syndrome may lead to chronic risks which the metabolic syndrome (MetS) can be mentioned [1].

MetS is a complex disorder characterized by a combination of several metabolic risk factors together with a series of symptoms, including abdominal obesity, dyslipidemia, hypertension, IR (with or without glucose tolerance), pre-inflammatory condition and hypercoagulable state [2]. Since this syndrome can cause cardiovascular diseases (CVDs) and diabetes, studying its root causes and preventions is of crucial importance. Due to differences in the diagnostic indicators, race and geographical features, MetS prevalence differs among the women suffering from PCOS throughout the world ranging from 7 to 43% [3, 4]. IR is one of the basics of PCOS and MetS pathophysiology [1].

IR is induced due to the oxidative stress-related mechanisms [5]. Diet has been recognized as one of the modifiable factors of the lifestyle with a significant impact on oxidative stress and IR [6]. Improper diet and sedentary lifestyle can undoubtedly cause and intensify the PCOS symptoms by aggravation of IR and serum insulin level along with increasing obesity [7]. Previous studies have addressed the role of some micronutrients in IR and its improvement [8].

Selenium (Se) is one of the essential minerals with antioxidant properties, which acts against the free oxygen radicals in reactive oxygen species (ROS) signaling pathways [9]. The inverse relation of Se and other antioxidants’ consumption with lower serum level of C-reactive protein (CRP) among the women suggests the anti-inflammatory role of this antioxidant nutrient. Moreover, high Se consumption can decline the risk of oxidative stress and inflammation-induced diseases through making changes in lipid metabolism [10].

The decreased serum level of Se was observed in PCOS patients [10]. Some studies have shown improvements in the insulin metabolism parameters and even triglyceride (TG) and very low-density lipoprotein (VLDL) level upon Se supplement prescription [11]. Other studies have reported a negative correlation between the dietary Se intake and salicylic acid (marker of inflammation) level and MetS [12]. However, Zagrodzki et al. found no significant difference in the Se serum level among PCOS patients and healthy subjects [13]. Hosseinzadeh et al. reported no desirable results from administration of Se supplement in PCOS patients. They declared that Se can even deteriorate the IR condition in these patients [14]. Surprisingly, Obeid et al. have mentioned a positive relationship between the Se content of the blood and MetS components [15].

Carotenoids are another class of micronutrients whose role in the improvement of oxidative stress has been well documented [16]. Ni et al. have shown the improving effect of carotenoids on IR [8]. Chromium (Cr) is a micronutrient, which can intensify the insulin effect. Hence, it is an essential element for glucose and lipid metabolism [17]. Cr supplements are reportedly able to improve insulin sensitivity and MetS parameters in type 2 diabetic patients [18]. Cr supplementation can have useful impacts on glycemic control and improvement of CVD risk and oxidative stress state in PCOS patients [19].

Zinc (Zn) is known as one of the basic components of superoxide dismutase enzyme and a cofactor for antioxidative enzymes [20]. This important element has a significant role in reducing IR and inflammatory markers [21].

The genes related to the regulation of vitamin D level have been proven to be involved in both lipid and glucose metabolism and blood pressure control [22]. Through preventing DNA damage at the cellular level, vitamin D may inhibit the inflammatory and oxidative stress processes [23]. Some studies have suggested the relationship between vitamin D and obesity, IR and hypertension among PCOS women [24, 25]. Vitamin D supplementation in PCOS women can also decrease the total testosterone (TT) and factors related to inflammation and oxidative stress and improve the IR and metabolic profiles [26]. Moreover, an inverse relationship has been observed between serum vitamin D and MetS components, including abdominal obesity, hypertension, and abnormal glucose homeostasis [27].

Antioxidant effects of vitamin E have been also proven [28]. The nutritional intake of this vitamin was lower in PCOS women in comparison with healthy subjects [29]. However, there are some controversies on the relation of this vitamin with MetS [30, 31].

To the best of knowledge, no study has addressed so far the nutritional intake of the above-mentioned micronutrients in PCOS patients with and without MetS, the present study aimed to investigate and compare their nutritional intake in these two mentioned groups of women.

## Methods

The present case-control study, which was accomplished from October 2016 to September 2017 in Tehran (Iran) on 42 PCOS patients, was approved by the Ethical Committee of Tarbiat Modares University (D525503). Indeed, it has been derived from an original cross-sectional study [32].

Among the PCOS patients who were investigated in the primary full study, 14 PCOS subjects with MetS were detected and considered as the case group. Also 28 randomly selected patients (twice the number of case group) who had PCOS but not MetS were considered as the control group. Since, we did not have more than 14 PCOS patients with MetS, for increasing the efficiency of the comparison, enhancement of the study accuracy, and decrease in the study errors statistically, we used a 2-fold control (28 PCOS patients without MetS). The two groups were matched in terms of age, BMI, physical activities, economic condition, and education level. The convenience sampling method was employed. The subjects were selected among the PCOS patients who referred to the gynecology or endocrine departments of the hospitals and private offices and met the inclusion criteria. All women were informed about the project and the written informed consent was obtained from all of them before participating in the study.

The inclusion criteria were Iranian race, diagnosis of PCOS, age range of 18–40 years, no medication with influence on MetS and PCOS components and appetite, no specific diet, and no pregnancy. The case group members should also have MetS in addition to the mentioned criteria. The Rotterdam criteria were used to verify the PCOS diagnosis (after the rejection of other androgen-increasing disorders). If two out of the following three observations were present, the participants were labeled as having PCOS:

- H [clinical (hirsutism) and/or biochemical hyperandrogenism (increased serum TT and/or free androgen index (FAI)]
- O [ovulation dysfunction (amenorrhea or oligomenorrhea)]
- P [PCOS sonographic view on ultrasound] [1].

MetS diagnosis was based on the NCEP ATP III criteria at the presence of the following three or more items [3]: waist circumference (WC) at least 95 cm in Iranian women [33], TG level of ≥150 mg/dl, high-density lipoprotein (HDL) < 50 mg/dl, blood pressure (BP) ≥130/85 mm-Hg, and fasting blood sugar (FBS) ≥ 110 mg/dl.

We asked the patients about their total revenues and then subtracted the expenditures incurred. These data were considered as economic levels after dividing by three for making tertiles. Thereafter, we assumed tertile 1 as level one, and so on. Eventually, economic level of the participants was classified into tertiles as follows: First level (low economic status), second level (medium economic status) and third level (high economic status). Physical activity of the participants was classified into the following three levels based upon the weekly schedule for exercise for at least 20 min per week: level 1 (none), level 2 (1–2 times/week), and level 3 (≥3 times/week).

A 147-item food frequency questionnaire (FFQ) was filled out for all participants. The reliability and validity of the questionnaire had been confirmed previously [34]. The questionnaire assesses the habitual diet over the past year. Participants were asked about their intake frequency for each food item consumed during the past year on a daily, weekly, or monthly basis; portion sizes of consumed foods reported in household measures were then converted to grams. In order to analyze food items in terms of the energy and micronutrient intake, designed Excel worksheet according to “Nutritionist 4 software data” (Iranian Food Composition Table and the food composition tables (FCT) of the United States Department of Agriculture (USDA)) was used. Energy intake of subjects was adjusted and then Z-score of the energy-adjusted intakes was computed. After that we removed out of range data in terms of energy (removed ±4SD of energy intake data). These dietary data allowed the calculation of the total intake per day. Using the obtained data, the amount of micronutrient intake (carotenoids, Zn, Se, Cr, vitamin D, and vitamin E) was determined for each participant.

Moreover, blood pressure and anthropometric measurements (height, weight, BMI and WC), hirsutism (as a clinical sign of hyperandrogenism) and menstrual regularity evaluations, ovarian ultrasonography and serum hormonal and metabolic parameter assays (to determine the amount of serum androgenic parameters and serum components of MetS) were performed.

### Clinical and laboratory evaluations

Hyperandrogenism, the fundamental yardstick in the diagnostic process of PCOS, appears as clinical demonstration and/or biochemical hyperandrogenemia. The Ferriman-gallwey score (FG- score) of 8 or more was defined as hirsutism (clinical hyperandrogenism) [32]. Hyperandrogenemia is characterized by an unusually high level of androgen produced by the ovaries or the adrenal glands [35]. Hyperandrogenemia was defined according to the cut-off determined for Iranian women [36]. TT and FAI were considered as diagnostic parameters for hyperandrogenemia. None of the participants had taken a medication interfering with their hormonal and metabolic profile in the last 3 months prior to the study.

TT and Sex Hormone Binding Globulin (SHBG) were measured by electro chemo-luminescence using Roche Company kit (Germany) and Kobas E 411 device (Germany). FAI was determined by dividing TT (nmol/lit) on SHBG (nmol/lit) multiplied by 100 [37]. TG and FBS were assessed by enzymatic-colorimetric method, and HDL was tested by immunoinhibition assay using Pars Company kits (Iran) and auto-analyzer device (BT2000) (Italy).

Ovarian volume larger than 10 cm^3^ in at least one ovary or observation of more than 5–8 small follicles was considered as positive PCOS. Menstrual cycle duration more than 35 days (oligomenorrhea) or more than 3 months (amenorrhea) was considered as positive ovulation dysfunction.

The minimum correlation coefficient, study power, and confidence interval were 0.5, 80 and 95%, respectively in the primary study. After data collection, they were analyzed by SPSS 16 software. Two-independent-samples T-test and Chi^2^ test were employed to compare the matching of the case and control groups. The Kolmogorov-Smirnoff’s (KS) test was used to check the normality of quantitative variables. In order to investigate the difference between the two groups, T-test and Mann-Whitney U test were performed for the normal and non-normal variables, respectively. *P* < 0.05 was considered significant.

## Results

According to Table 1, the two groups were not significantly different in terms of age, BMI, education level, economic level and physical activity (*P* > 0.05).

**Table 1:** Comparison of demographic components between the case and control groups

Demographic components	PCOS with MetS *n* = 14	PCOS without MetS *n* = 28	*P*-value
Age (year)	31.28 ± 4.87	29.42 ± 4.67	0.21*
BMI (Kg/m^2^)	29.92 ± 3.71	30.19 ± 3.22	0.80*
**Physical activity**			
No physical activity	9 (33.3)	18 (66.7)	0.32**
1–2 times a week	3 (60)	2 (40)	
> 3 times a week	2 (20)	8 (80)	
**Education level**			
High school and diploma	3 (21.4)	12 (42.9)	0.34**
Bachelor and technician	8 (57.1)	11 (39.3)	
Higher than bachelor	3 (21.4)	5(17.9)	
**Economic condition**			
Low economic status	5 (35.7)	13 (46.4)	0.20**
Medium economic status	4 (28.6)	11 (39.3)	
High economic status	5 (35.7)	4 (14.3)	

*PCOS* Polycystic Ovary Syndrome, *MetS* Metabolic Syndrome, *BMI* Body Mass Index

*P-values related to t-Test

** *P*-values related to Chi-square test

Data are given as N (%) except for age and BMI (Mean ± SD)

Statistical significance was set at *P* < 0·05

Table 2 presents the results of the comparison between the case and control groups in terms of PCOS diagnostic criteria, MetS components and IR indicators. As shown, the FG-score of hirsutism was significantly higher in the case group (*P* = 0.01). TT and FAI were significantly higher in the case group comparing to the control group (*P* < 0.001 and *P* = 0.005, respectively). Among the MetS components, HDL, TG, systolic blood pressure (SBP), and diastolic blood pressure (DBP) showed a significant difference between the two groups. HDL was significantly lower (*P* = 0.001), and TG (*P* < 0.001), SBP (*P* = 0.002) and DBP (*P* = 0.011) were significantly higher in the case group than in the control group. FBS and WC of the two groups, however, showed no significant difference (*P* > 0.05). Fasting insulin and the homeostatic model assessment of insulin resistance index (HOMA-IR) were significantly higher in the case group comparing to the control group, indicating the higher IR among the case group members (*P* = 0.041 and *P* = 0.027, respectively). The quantitative insulin sensitivity check index (QUICKI) was significantly lower in the case group, reflecting the lower insulin sensitivity in these participants (*P* = 0.025).

**Table 2:** Comparison of basic PCOS, MetS and insulin resistance diagnostic criteria between the case and control groups

PCOS diagnostic component	PCOS with MetS n = 14	PCOS without MetS n = 28	*P*-value
Hirsutism score (FG-Score)	9.14 ± 3.65	5.53 ± 4.25	0.01*
Number of ovarian follicle	10.64 ± 4.03	12.25 ± 3.06	0.25*
Menstruation duration (day)	56.57 ± 31.4	52.53 ± 29.26	0.72*
TT (nmol/l)	3.42 ± 3.02	1.09 ± 0.61	< 0.001**
FAI	9.91 ± 9.57	3.85 ± 3.53	0.005**
SHBG (nmol/l)	37.67 ± 13.47	39.88 ± 19.60	0.70**
**Metabolic syndrome components**			
FBS (mg/dl)	92.21 ± 8.45	91.46 ± 9.04	0.10**
HDL-C (mg/dl)	39.35 ± 9.73	49.6 ± 8.90	0.001**
TG (mg/dl)	192.21 ± 51.20	108.36 ± 44.88	< 0.001**
WC (cm)	95.92 ± 10.99	92.12 ± 11.14	0.30**
SBP (mmHg)	128.86 ± 25.74	110.68 ± 10.74	0.002**
DBP (mmHg)	82.57 ± 24.46	70.25 ± 11.12	0.01*
**Insulin resistance indices**			
Fasting insulin (μU/ml)	22.71 ± 17.49	14.45 ± 8.0	0.04**
HOMA-IR	5.25 ± 3.68	3.28 ± 1.90	0.02 **
QUICKI	0.30 ± 0.02	0.32 ± 0.02	0.02**

*PCOS* Polycystic Ovary Syndrome, *MetS* Metabolic Syndrome, *FG-Score* Ferriman-Gallwey score, *TT* Total Testosterone, *FAI* Free Androgen Index, *SHBG* Sex Hormone Binding Globulin, *FBS* Fasting Blood Sugar, *HDL-C* High Density Lipoprotein Cholesterol, *TG* Triglyceride, *WC* Waist Circumference, *SBP* Systolic Blood Pressure, *DBP* Diastolic Blood Pressure, *HOMA-IR* Homeostatic Model Assessment of Insulin Resistance, *QUICKI* Quantitative Insulin Sensitivity Check Index

Data are presented as mean ± SD. *P-values refer to Mann-Whitney test. **P-values refer to t-Test. *P* < 0.05 was considered significant

Results in Table 3 show that the dietary intake of Se (*P* < 0.001), Cr (*P* = 0.006), Zn (*P* = 0.038), carotenoids (*P* < 0.001), and vitamin E (*P* < 0.001) was significantly lower in the case group comparing to the control group. However, the dietary intake of vitamin D showed no significant difference between the two groups (*P* > 0.05).

**Table 3:** Comparison of the nutritional intake of the micronutrients between the case and control groups

Micronutrient	PCOS with MetS n = 14	PCOS without MetS n = 28	*P*-value
Se (μg/day)	37.52 ± 28.15	110.42 ± 49.93	< 0.001
Cr (μg/day)	0.03 ± 0.08	0.11 ± 0.08	0.006
Zn (mg/day)	6.76 ± 6.43	10.46 ± 4.60	0.03
Carotenoid (mg/day)	2.01 ± 8.66	15.78 ± 9.80	< 0.001
Vitamin D (μg/day)	2.58 ± 1.57	3.13 ± 1.40	0.25
Vitamin E (mg/day)	6.17 ± 7.20	17.21 ± 7.02	< 0.001

*PCOS* Polycystic Ovary Syndrome, *MetS* Metabolic Syndrome, *Se* Selenium, *Cr* Chromium, *Zn* Zinc

Data are reported as mean ± SD

*P*-value related to t-Test

*P* < 0.05 was considered significant

## Discussion

The results of the present study indicate that the nutritional intake of Se, Cr, Zn, carotenoids and vitamin E was lower in the PCOS patients with MetS (case group) compared with those without MetS (control group). The IR in the case group was significantly higher than in the control group.

IR is the fundamental etiology of PCOS [1]. It is also a key factor in the pathophysiology of MetS whose effect on this syndrome has been proven [38]. The presence of some changes in the IR specific indicators, which illustrates increased IR in the case group, can be justified due to the severity of the metabolic complications of these patients comparing to the non-MetS patients.

Oxidative stress can result in IR through various molecular mechanisms [5]. Se is one of the essential antioxidants, which can protect the cells against oxidative stress by expression of selenoprotein genes and anti-inflammatory mechanism [39]. According to the literature review, no similar study has been conducted on comparing the dietary intake of micronutrients in PCOS patients with and without MetS. Therefore, we inevitably used the studies, which had investigated these micronutrients separately.

Reduction in the serum level of Se was observed in PCOS patients [10]. In a systematic review in 2019 in Iran, the decreased serum level of Se was found in PCOS patients. Only one-fourth of the reviewed papers reported the protective impact of Se supplement on IR; Two third of them, however, proved the antioxidant effect of Se. In the above study, the papers addressing the anti-androgenic effects of Se showed contradicting results. It was also indicated that Se can reduce dyslipidemia and IR. The authors eventually declared that the available data are not sufficient to support the protective effect of Se in PCOS patients [40]. Gregorio et al. expressed that application of antioxidants, especially via nutrition, can improve MetS, and that lifestyle change can be an effective approach in the treatment of these patients [41]. Some studies considered Se supplement administration as an effective method in MetS patients [42]. Zulet et al. (2009), investigating the relation between Se nutritional intake and serum level of salicylic acid as an inflammatory marker of MetS components, found out lower salicylic acid levels in women receiving more nutritional Se. They believed that nutritional Se has anti-inflammatory potential, which can be effective in MetS patients [12]. In contrary, some studies have reported the impact of increased serum level of Se on MetS enhancement [43]. Ford et al. conducted a cross-sectional study in 2003 in the US and evaluated the antioxidant conditions among MetS patients. The results indicated that adult MetS patients had lower concentrations of several antioxidants, which can justify the increased risk of diabetes and CVDs among them; however, they showed no significant difference in the serum Se level [44]. Ghayour-Mobarhan et al. (2008) studied the obese MetS women in UK and realized that their serum Se level and nutritional intake of Se were higher than those in healthy women [45].

The contradiction in the results of various studies in terms of Se relationship with MetS could be due to diverse study design, difference in the studied population, racial diversity, and fundamentally, the complex nature of both MetS and PCOS. Moreover, the design of the present study is different from that of the mentioned researches, and the studied population was selected from among PCOS patients and not healthy women, which can be effective on the difference in the results. Furthermore, we investigated the dietary intake of the mentioned micronutrients, not their serum level. Since the majority of the previous studies have addressed the serum level rather than the dietary intake, we inevitably compared our results with these studies.

The current research results also indicated that the dietary intake of Cr was lower in the PCOS patients with MetS when compared with those without MetS. Similarly, another research showed that 8 weeks of Cr application in infertile women with PCOS could have a positive impact on glycemic control, decline of fasting insulin, HOMA-IR, TG, VLDL and total cholesterol, increase of antioxidant capacity, and improvement of oxidative stress [19]. A review article stated that Cr supplement has limited impact on the weight loss, glucose control, lipid profile and hormonal disturbance of PCOS women [46]. Cr has a high antioxidant potential [47]. Due to the insulin-mimetic effects of Cr and Se, these two elements have been prescribed to improve MetS [42]. Regarding the etiologic background of oxidative stress in MetS [48] and PCOS, dietary deficiency of this antioxidant micronutrient (Cr) in people suffering from both MetS and PCOS seems rational in comparison with those only having PCOS. Hence, low dietary intake of Cr could be related to the deterioration of their metabolic and hormonal condition.

In a meta-analysis conducted by Abedini et al. in 2019, it was revealed that the serum level of Zn is lower in women with PCOS when compared to the control group [49]. In conformity with the present study, Freitas et al. observed lower nutritional intake of Zn in people with MetS when compared with healthy subjects [50]. In contrary, Fang and colleagues observed no relationship between the serum level of Zn and MetS and its components [43]. In the present study, it was revealed that the dietary intake of Zn was lower in the PCOS patients with MetS. Therefore, if it cannot be considered as one of the definite involving factors in MetS development in PCOS patients, it can be probably effective in deterioration of the metabolic condition of these patients. This can be due to the antioxidant function of Zn and the related epigenetic modulations. The study of Fang et al. was performed on Chinese men and women over 40 years old while the present study was performed on women with PCOS. The effect of Zn on the insulin signaling mechanisms has been proven [51]. Studies have revealed that IR in PCOS patients is the consequence of a post-receptor defect in insulin performance [52]. Regarding the etiologic basis of IR in these patients and considering the role of Zn in insulin signaling mechanisms, the difference between the results of this study with that of Fang et al. could be justified by the fact that in PCOS patients, the effect of Zn and its dietary intake on the metabolic status and components of MetS, which is also linked to insulin resistance, is more pronounced.

Studies have suggested the improving effect of some carotenoids on IR [8]. Researchers have related the lower serum level of some carotenoids with insulin sensitivity deficit and declared that long-term deficiency of beta-carotene can predict type 2 diabetes [53]. Some other carotenoids can decline the cardio-metabolic risk factors and visceral fat via modulating the expression of nuclear transcription factors such as NF-kB [54]. Other studies have mentioned the importance of lipid-soluble carotenoids in MetS improvement due to their antioxidant and anti-inflammatory effects [55]. In 2019, it was shown that carotenoids are inversely related to MetS [56]. Mei et al., however, observed no relation between the dietary intake of carotenoids and MetS in 2015 [31]. In 2018, a study showed that crocetin carotenoid can decrease the PCOS induced by dihydrotestosterone in rats [57]. In the present study, the dietary intake of carotenoids was significantly lower in the PCOS patients with MetS when compared to the control group. Due to the antioxidant and anti-inflammatory potential of carotenoids, dietary intake deficiency of these micronutrients can be effective in the worsening of the metabolic condition of PCOS patients.

Low vitamin E intake was observed in PCOS patients [29]. Administration of dietary supplement of vitamin E can have positive impacts on the condition of these patients [58–60]. Wei et al. observed no relationship between the nutritional intake of vitamin E and MetS [31]. However, Kim et al. declared the protective effect of vitamin E provided by food on MetS, which can be due to the antioxidant property of this vitamin [30]. Low nutritional intake of alpha-tocopherol (a type of antioxidant vitamin E) has been related to IR [53]. Goncalves et al. (2017) also observed the effectiveness of vitamin E in prevention from MetS [54].

In this study, the shortage of dietary intake of vitamin E was observed in the PCOS patients with MetS. The difference between our study and that of Wei et al. could be due to race and population study differences, as well as the different design of these two studies. Wei et al. studied men and women over 18 years old while the subjects of this study suffered from an insulin-resistance disease (PCOS), and in the case group, this was exacerbated by the presence of MetS. Therefore, lower levels of carotenoid and vitamin E in the diet of PCOS patients (comparing to healthy subjects in terms of IR) could be more effective in the emergence of MetS and deterioration of their metabolic condition.

The present study enjoys some valuable strengths. It is the first research that compares the dietary intake of micronutrients in PCOS patients with and without MetS. Elimination of confounding factors via matching the two groups, employing precise inclusion criteria, and application of the reliable and valid 147-item FFQ are among the other strong points of this study. This is, however, a case-control study, and hence, it could not accurately define the cause-effect relationship. Time-consuming nature of the 147-item FFQ and difficulty in remembering and estimating the dietary intake in the last year can be mentioned as the limitations of the present study. Nevertheless, we excluded the estimations with standard deviation (SD) above and below than 4.

According to the literature review, since, no study has addressed the nutritional condition of PCOS patients with and without MetS; therefore, we had to select the similar studies carried out on PCOS and MetS patients separately to compare their finding with ours. It seems that further cohort studies with higher population and clinical trials are required for more definite results. This study also did not investigate the serum level of the studied micronutrients, which could be helpful for further scientific richness of the present research.

## Conclusion

Since the results of the present study showed that PCOS patients without MetS had more intakes of aforementioned micronutrients than those with MetS, hence, it can be concluded that the dietary intakes of these nutrients could probably have protective effects on MetS.

## Data Availability

The data sets used and analyzed during the current study are available from the corresponding author on reasonable request.

## Abbreviations

PCOS: Polycystic Ovary Syndrome
MetS: Metabolic Syndrome
BMI: Body Mass Index
FG-score: Ferriman-Gallwey score
TT: Total Testosterone
FAI: Free Androgen Index
SHBG: Sex Hormone Binding Globulin
FBS: Fasting Blood Sugar
HDL-C: High Density Lipoprotein Cholesterol
TG: Triglyceride
WC: Waist Circumference
SBP: Systolic Blood Pressure
DBP: Diastolic Blood Pressure
HOMA-IR: Homeostatic Model Assessment of Insulin Resistance
QUICKI: Quantitative Insulin Sensitivity Check Index
Se: Selenium
Cr: Chromium
Zn: Zinc
